# Mitochondrial DNA sequence variants in epithelial ovarian tumor subtypes and stages

**DOI:** 10.1186/1477-3163-6-1

**Published:** 2007-01-26

**Authors:** Felix O Aikhionbare, Sharifeh Mehrabi, K Kumaresan, Mojgan Zavareh, Moshood Olatinwo, Kunle Odunsi, Edward Partridge

**Affiliations:** 1 Department of Medicine, Morehouse School of Medicine, Atlanta, GA 30310, USA; 2 Department of Obstetrics and Gynecology, Morehouse School of Medicine, Atlanta, GA 30310, USA; 3 Department of Gynecologic Oncology, Roswell Park Cancer Institute, Buffalo, NY 14263, USA; 4 University of Alabama, Comprehensive Cancer Center, Birmingham, AL 35294, USA

## Abstract

**Background:**

A majority of primary ovarian neoplasms arise from cell surface epithelium of the ovaries. Although old age and a positive family history are associated risk factors, the etiology of the epithelial ovarian tumors is not completely understood. Additionally, knowledge of factors involved in the histogenesis of the various subtypes of this tumor as well as those factors that promote progression to advanced stages of ovarian malignancy are largely unknown. Current evidence suggests that mitochondrial alterations involved in cellular signaling pathways may be associated with tumorigenesis.

**Methods:**

In this study, we determined the presence of polymorphisms and other sequence variants of mitochondrial DNA (mtDNA) in 102 epithelial ovarian tumors including 10 matched normal tissues that paired with some of the tumors. High-resolution restriction endonucleases and PCR-based sequencing were used to assess the mtDNA variants spanning 3.3 kb fragment that comprised the D-Loop and 12S rRNA-tRNA^phe^, tRNA^val^, tRNA^ser^, tRNA^asp^, tRNA^lys^, *ATPase 6, ATPase 8, cytochrome oxidase I and II *genes.

**Results:**

Three hundred and fifty-two (352) mtDNA sequence variants were identified, of which 238 of 352 (68%) have not been previously reported. There were relatively high frequencies of three mutations in the 12S rRNA gene at np 772, 773, and 780 in stage IIIC endometrioid tumors, two of which are novel (773delT and 780delC), and occurred with a frequency of 100% (7/7). Furthermore, two mutations were observed in serous tumors only at np 1657 in stage IV (10/10), and at np 8221delA in benign cystadenomas (3/3) and borderline tumors (4/4). A high frequency, 81% (13/16) of TC insertion at np 310 was found only in early stages of serous subtype (benign cystadenomas, 3/3; borderline tumors, 4/4; stage I tumors, 2/5 and matched normal tissues 4/4).

**Conclusion:**

Our findings indicate that certain mtDNA mutations can reliably distinguish the different histologic subtypes of epithelial ovarian tumors. In addition, these data raise the possibility that certain mtDNA mutations may be useful biomarkers for predicting tumor aggressiveness and may play a potential role in tumorigenesis.

## Background

Epithelial ovarian cancer is the fifth leading cause of cancer mortality among women in the United States [1]. The majority (80–90%) of benign and malignant ovarian tumors originate from the surface epithelium, even though all cell types of the human ovary may undergo neoplastic transformation [2,3]. Most of the histopathological differences that are reflected as serous, endometrioid, mucinous, clear cell, and transitional cell types of ovarian cancer cannot be clearly explained by the presence or absence of specific genetic changes [4]. Ovarian cancer is notoriously difficult to diagnose in its early stages. Consequently, approximately 90% of the patients with epithelial ovarian cancer are diagnosed with metastasis to the pelvis and upper abdomen and, for these patients, five year survival rates are less than 30% [1]. In contrast, the small proportion of patients diagnosed with stage I ovarian cancer (confined to the ovaries) have a five year survival rate in excess of 90% [1]. Even when patients with the same stage and histologic type of ovarian cancer are considered, the biologic factors that could predict disease behavior are not well understood. Thus, there is a pressing need to identify ways to distinguish and understand the different epithelial ovarian tumor subtypes in order to develop effective treatment strategies.

Human mitochondrial gene mutations have increasingly been associated with various cancers but with unclear pathophysiological significance [3,5-9]. Most mtDNA mutations occur in coding sequences because mtDNA lacks introns. The mitochondrial genome is more vulnerable to oxidative damage and undergoes a higher rate of mutation than does the nuclear genome [10,11]. Some of the mitochondrial genome aberrations found in cancer tissues are mutations in mtDNA-encoded Complexes I, III-V as well as mutations in the hypervariable regions [9]. MtDNA mutations in tumor cells are consistent with previous reports that tumor cells are subject to constitutive oxidative stress [9,11,12]. Although most of the reported mtDNA mutations in cancer are homoplasmic polymorphisms which are considered too subtle to cause any effect on oxidative phosphorylation (OXPHOS), long-term accumulation of subtle difference in OXPHOS activity may eventually result in oxidative stress [12,13]. Consequently, variations in the mtDNA may potentially play a role as a modifying risk factor in the development of age related diseases such as ovarian cancer. Mitochondrial aberrations have been identified in tumors of colorectal, breast, head and neck, kidney, lung, stomach and in the hematologic malignancies, leukemia and lymphoma [7,13-20]. Hence, we sought to determine the impact of mtDNA sequence variants in the pathogenicity of epithelial ovarian tumor subtypes.

Here we report the identification of mtDNA sequence variants in multiple specimens of ovarian carcinoma using a high-resolution restriction endonucleases and PCR-based sequencing analyses. We have found several highly frequent mtDNA sequence variants that could differentiate the three major histopathological subtypes and stages of serous, endometrioid and mucinous carcinomas of the ovary.

## Methods

### Subjects

One hundred and two frozen epithelial ovarian tumor tissues from three histologic subtypes (serous n = 42; endometrioid, n = 33; mucinous n = 17), each of the stages I-IV, benign cystadenomas, borderline tumors and 10 matched normal tissues that paired with the tumors were obtained from Southern Regional Cooperative Human Tissue Network and University of Alabama-Birmingham (UAB)-Ovarian Spore Center. The ovarian tumor subtypes and stages were histopathologically determined on the basis of the criteria outlined by the International Federation of Gynecology and Obstetrics (FIGO). All studies were implemented under Morehouse School of Medicine and UAB Institutional Review Board approval protocols. MtDNA was isolated from the frozen tissues using centrifugation method according to the manufacturer's protocols (BioVision, Research Products). Total mtDNA was quantified and diluted to 50 ng/μl for PCR reaction.

### High Resolution Restriction Endonucleases and PCR-based Analysis of mtDNA variants

Extracted mtDNA from each tissue sample was PCR amplified using two large sets of overlapping primers. The first primer sets (*for-5'-CCGGGCCCATAACACTTGGG-3'*, position 16,453→16,472 in MITOMAP and *rev-5'-GGAGTGGGTTTGGGG CTAGG-3' *1,696→1,677) yielded 1.8 kb fragment that flank the D-loop and part of 12 rRNA regions and the second primer sets (for-*5'-GGATGCCCCCCACCCTACC-3'*, 7,392→7,410 and *rev-5'-CCTTGTGGTAAGAAGTGGGC-3'*, 8921-8902) yielded 1.5 kb fragments that spanned part of cytochrome oxidases I, II (COX I and II), ATPase 8 and ATPase 6 regions, which have been previously described [21,22]. The tRNAs enclosed in these regions are: Phe, Val, Ser, Asp, and Lys. The PCR was performed with 35 cycles of denaturation at 94°C for 1 minute, annealing at 59°C for 1 minute and extension at 72°C for 1 minute, and with the second set of primers, the annealing was 62°C for 1 minute. Amplified fragments were then purified using the QIAquick (QIAGEN) gel extraction kit. Each PCR product was digested with 14 restriction endonucleases (*AluI, AvaII, BamHI, DdeI, HaeII, HaeIII, HhaI, HincII, HinfI, HpaI, MspI, MboI, RsaI, and TaqI*) and were electrophoresis-gel analyzed side-by-side. Any mtDNA PCR fragments showing differences in banding patterns between subtypes, stages of tumor and matched normal tissue samples were first directly sequenced to identify the exact nature of the new length variants detected by restriction analysis. Additionally, all the amplified products were sequenced with the same PCR primer sets, to assess the presence of other mtDNA mutations within the samples. PCR products were sequenced using ABI PRISM 310 Genetic Analyzer (Perkin-Elmer, Foster, CA).

### Analysis of the mtDNA Sequences

The results of the mtDNA sequence analysis were compared with the published NCBI sequence (Accession # J01415). Furthermore, the sequence variants were compared with those in the mtDNA databank [3] to verify if the changes detected have previously been reported. Sequence alignment among subtypes and within stages were performed using Vector NTI advance 10-Align X program (Invitrogen). MtDNA sequence variants present in both the tumor and matched normal tissue were scored as germ-line variants. Any mtDNA sequence variant that were different between the tumor and the matched normal tissues were scored as somatic mutations. All sequence variants were confirmed by repeated analysis of the mtDNA extracted from the study samples.

## Results and Discussion

**Figure 1:**
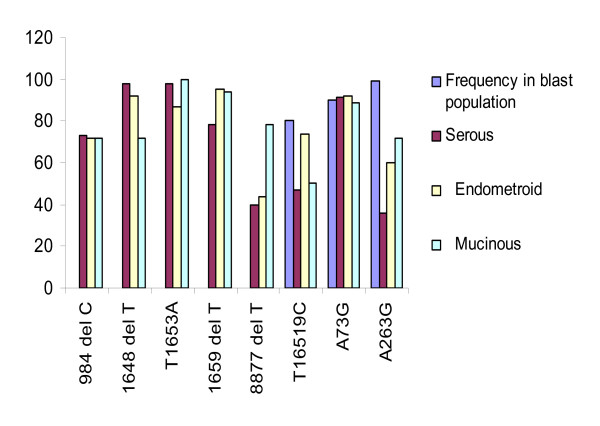
Distributions of the most frequent mtDNA mutations (984delC-*12S rRNA*; 1648delC, T1653A, 1659delT-*tRNA*^*val*^; 8877delC-*ATPase 6*; T16519C, A73G, A263G-D-loop region) in three epithelial ovarian tumor subtypes compared to 100 human genome sequences [24]. Mutation A263G appears to be less frequent in the epithelial ovarian tumor subtypes compared to the general population and the mtDNA-*tRNA*^*val *^1653delT variant may be considered as a causative event for the three epithelial ovarian tumor subtypes.

The restriction analyses identified a number of band shifts indicating site gains or losses between and among ovarian subtypes and stages. However, these bands only indicated that the predicted sequence had changed. They did not identify specific base pair changes. We, therefore, used the original primers to sequence the band shifts in order to identify these specific changes. Among 102 ovarian tumor samples (including 10 paired tumor and matched normal tissues) that were sequenced, a total of three hundred and fifty-two (352) mtDNA variants were observed over a span of 3.3 kb fragment, including D-Loop, 12S rRNA-tRNA^phe^, tRNA^val^, COX I, tRNA^ser^, tRNA^asp^, COX II, tRNA^lys^, *ATPase 6 *and *ATPase 8 *(see additional file 1) and 238 of 352 (68%) were not previously reported [23]. Insertions and deletions accounted for 38.3% (135/352) of the observed mtDNA sequence variants suggesting instability of mtDNA in epithelial ovarian tumors and is consistent with previous studies by Gomez-Zaera *et al*., [6] and Vega *et al*., [19]. A high frequency of mtDNA mutations among our study samples, which have been previously reported [23] were apparent at np A263G (93/102), A1438G (95/102), A8860G (96/102) as shown in Table 1 and additional file 1. Also, there were high incidences (86–100%) 1648delT, T1653A/delT, 1659delT mtDNA sequence variants among the three epithelial ovarian tumor subtypes and stages compared with 100 human genome sequences [24] (Figure 1). MtDNA sequence variants T16519C, A73G and A263G were found at frequencies of 36%–95% among our samples and these variants have been observed in various ethnic groups and other cancers [25]. A polymorphism, T16519C, has previously been shown to significantly increase breast cancer risk [26].

**Table 1:** High frequency mtDNA variants among three epithelial ovarian tumor subtypes

Mitochondrial genes/regions	Nucleotide Position np	Mutation Frequency **Total # of cases 102**	% frequency in subtypes		
			
			Serous	Endometrioid	Mucinous
D-loop	16487 **del A**^1^	73	87	69	39
D-loop	T16519C	52	47	74	50
D-loop	A73G	59	91	92	89
D-loop	A263G	93	36	60	72
12S rRNA	**984 del C**^1^	**58**	**73**	**72**	**72**
	**C984G**^1^	**16**			
12S rRNA	A1438G	95	91	95	94
tRNA^val^	1648 **del T**^1^	93	98	92	72
tRNA^val^	**1653 del T**^1^	**20**	**98**	**87**	**100**
	**T1653**A^1^	**76**			
tRNA^val^	1659 **del T**^1^	89	78	95	94
COX2	8237 **del A**^1^	68	82	49	67
ATP6	A8860G	96	96	97	83
ATP6	8877 **del T**^1^	49	40	44	78
ATP6	**T8889 A**^1^	**51**	**93**	**87**	**83**
	**T8889C**^1^	**16**			
	**8889del T**^1^	**24**			

^1 ^Not reported in mtDNA databank.

The frequency % of the three subtypes at np 984,1653 and 8889 represents the sum total of **the different types **of mutations occurring at that position.

**Figure 2:**
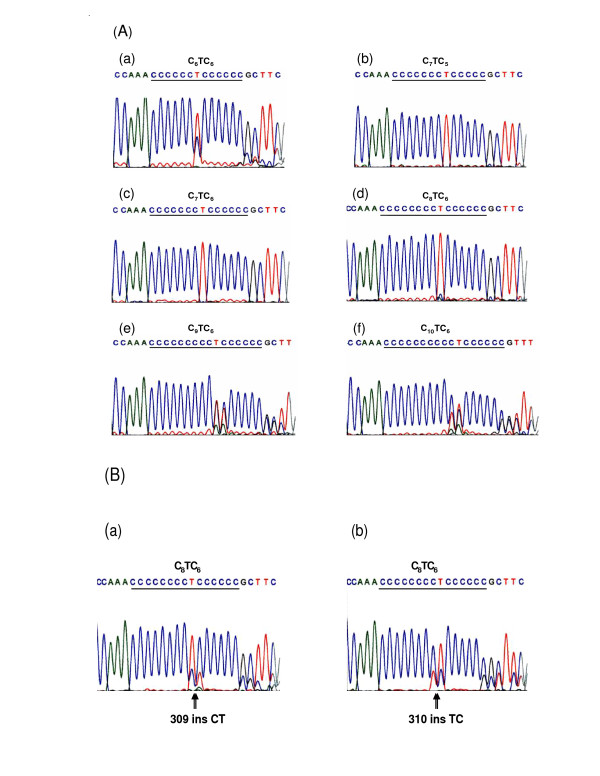
A. MtDNA sequence electropherograms showing variations in consecutive C-stretch at np 303–310 of the D-loop region obtained from the three subtypes of the epithelial ovarian neoplasms (a) C_6_TC_6 _and (b) C_7_TC_5 _sequences from stage I of endometrioid tumor; (c) C_7_TC_6 _and (d) C_8_TC_6 _sequences from stage I of mucinous tumor; (e) C_9_TC_6 _sequence obtained from stage III of mucinous tumor; (f) C_10_TC_6 _sequence from stage III of serous tumor. Interestingly, the C-stretch instability at np 303–315 was observed in 97% of our study samples. B. Sequence electropherograms showing the mtDNA instability at np (a) 309insCT in stage II of endometrioid tumor and (b) 310insTC observed only in early stages of serous subtype in this study. Arrow shows the unique 309insCT and 310insTC patterns respectively.

Consistent with other studies, we have observed the incidence of the polynucleotide repeat sequence at np 303–315, which has already been reported as a mutational hotspot D310, in a wide variety of human neoplasms [14,27-32]. The number of cytosine residues in the first stretch varied from C6-10T followed by the second stretch of C5-6 (Figure 2A). The reference sequence (mitomap-J01415) of this hot spot was C7TC5 and that of the general population with NCBI Blast Search was C7-8TC6 [33].

The c-stretch instability at np 303–315 was observed in 97% of the study samples, including the 10 tumor samples that paired with the matched normal tissues. This finding suggests that the observed c-stretch variants in ovarian tumors are germline origin. The instability at np 303 has been observed in some premalignant lesions of head and neck [16] and early stages of ovarian carcinomas [34], although none of the associations in those studies reached statistical significance. Perhaps the most striking observation was the incidence of TC insertion at np 310 found at a relatively high frequency of 81% (13/16) only in the early stages of serous subtype, which included benign cystadenomas 3/3, borderline tumors 4/4, stage I tumors 2/5, and matched normal tissues 4/4. While we could not conclusively rule-in poly C and the TC insertion instabilities as a major factor in the predominant subtype of epithelial ovarian tumorigenesis, certainly, the weight of the evidence, in spite of the limited sample size was not against it.

Different mechanisms have been put forward to explain the genomic instability of this stretch of cytosine residues [34-37]. The relative proportion of variable length polyC tracts appears to be actively maintained during cell division despite evidence of random mtDNA segregation, suggesting denovo regeneration of specific pattern following cell division by unknown molecular mechanisms [38,39]. It is unclear if this instability is associated with ethnicity, heredity or tissue specificity. Therefore, it is tempting to explore the significance of this D310 hot spot as a biomarker in detection, differentiation and progression of the various subtypes and stages of ovarian tumors.

Interestingly, we did observe three mtDNA mutations in the 12S rRNA gene at np A772T, 773delT, and 780delC in endometrioid stage III tumors, of which 773delT (7/7) and 780delC (7/7) with a frequency of 100% in endometrioid stage IIIC has not been reported. Furthermore, two mutations of interest were observed only in serous subtype, at np 1657delC in stage IV (10/10) and 8221delA in benign cystadenomas (3/3) and borderline tumors (4/4). To our knowledge, the incidence of specific mtDNA variants in association with FIGO stages and subtypes of epithelial ovarian tumor have not been reported.

A previous study by Liu *et al*., [40] reported a high incidence of somatic mtDNA mutations in ovarian carcinoma and it is consistent with our observation where we observed somatic mutations in the mtDNA of a majority of the tumor samples. In contrast to the same study [40], we did not find an increase or decrease of CA repeats at 514–524, except few ins/del sequence variants with no significant association with specific ovarian tumor stages, subtypes or matched normal tissues. Perhaps the discrepancies may be attributed to the histopathologic stages of the tumor tissues that we analysed and the mtDNA haplogroup within ovarian cancer.

## Conclusion

Notably, most of the identified mtDNA sequence variants occurred in D-loop region and any mutation in this region may potentially modify the rate of mtDNA replication. The D-loop region is important for replication and expression of mitochondrial genome due to the leading-strand origin of replication and the promoters of transcription [41]. Although our study lacks functional data and clinical outcome of the mitochondrial genome alterations in relationship to ovarian neoplasms, it suggests that mtDNA sequence variants may play a role in etiological differences that may exist between the pathogenicity of subtypes and stages of benign and invasive epithelial ovarian tumors. Large population-based studies are required to precisely quantify the functional role of mtDNA sequence variants in histological subtypes and stages of ovarian cancer. Given that we only sequenced 3.3 kb of 16.5 kb fragment of mitochondrial genome, the level of mtDNA sequence variants that could be correlated with ovarian tumor subtypes and stages may far exceed the number we have observed in this study.

## Competing interests

The author(s) declare that they have no competing interests.

## Authors' contributions

FOA conceived, designed, coordinated the study, and participated in data analysis and drafted the manuscript. MS participated in acquisition and analysis of the data and helped to draft the manuscript. KK participated in acquisition and analysis of the data and helped to draft the manuscript. MZ participated in acquisition of the data. MO participated in the review of the manuscript. KO helped to draft the manuscript. EP provided some of the ovarian tumor samples and participated in the review of the manuscript. All authors read and approved the final manuscripts.

## Supplementary Material

Additional file 1 Mitochondrial DNA mutations in epithelial ovarian tumors. The data provided represent the mitochondrial sequence variants spanning 3.3 kb fragment that comprised the D-Loop and 12S rRNA-tRNA^phe^, tRNA^val^, tRNA^ser^, tRNA^asp^, tRNA^lys^, *ATPase 6, ATPase 8, cytochrome oxidase I and II *genes. in our study epithelial ovarian tumor samples. Click here for file
