# Supplementary material for: Mitochondrial DNA sequence variants in epithelial ovarian tumor subtypes and stages
Source: J Carcinog. 2007 Jan 26;6:1. doi: 10.1186/1477-3163-6-1 (PMC1794240; doi:10.1186/1477-3163-6-1)
Supplement: Additional file 1 — Mitochondrial DNA mutations in epithelial ovarian tumors. The data provided represent the mitochondrial sequence variants spanning 3.3 kb fragment that comprised the D-Loop and 12S rRNA-tRNAphe, tRNAval, tRNAser, tRNAasp, tRNAlys, ATPase 6, ATPase 8, cytochrome oxidase I and II genes. in our study epithelial ovarian tumor samples. [file 1477-3163-6-1-S1.doc]

Additional File 1. mtDNA mutations in epithelial ovarian tumors

Mutation Mt. Nucleotide Frequency

Positions Gene Change Total -102

16486 D-loop Del A 9

16487 D-loop Del A  **73**

16496 D-loop G→C 4

16497 D-loop A→G* 4

16504 D-loop G→T 2

16505 D-loop Ins C 5

16508 D-loop Ins C 11

16509 D-loop Ins C 7

16509 D-loop T→C 3

16512 D-loop T→C 3

16519 D-loop T→C*  **52**

16527 D-loop C→T 3

64 D-loop C→T* 3

72 D-loop T→C* 5

73 D-loop A→G*  **59**

93 D-loop A→G* 5

114 D-loop C→T* 2

143 D-loop G→A* 4

146 D-loop T→C* 14

150 D-loop C→T* 15

151 D-loop C→T* 3

152 D-loop T→C* 34

153 D-loop A→G* 1

182 D-loop C→T* 7

183 D-loop A→G* 3

185 D-loop G→A* 3

185 D-loop G→T* 1

189 D-loop A→C* 3

189 D-loop A→G* 8

194 D-loop C→T* 2

195 D-loop T→C* 47

195 D-loop T→A* 1

198 D-loop C→T* 7

199 D-loop T→C* 3

200 D-loop A→G* 9

204 D-loop T→C* 5

204 D-loop T→G* 1

207 D-loop G→A* 4

215 D-loop A→G* 1

234 D-loop A→G* 3

235 D-loop A→G* 1

236 D-loop T→C* 2

244 D-loop A→G 5

247 D-loop G→A* 6

249 D-loop A→G* 3

260 D-loop G→A* 1

263 D-loop A→G* **93**

295 D-loop C→T* 4

297 D-loop A→G* 3

309 D-loop Ins TT 1

309 D-loop Ins CC* 4

309 D-loop Ins C 11

309 D-loop Del C* 1

309 D-loop Ins T 29

309 D-loop Ins CT 10

310 D-loop Ins C* 1

310 D-loop Ins TC 16

310 D-loop T→C* **46**

310 D-loop Del T 1

315 D-loop Ins A 1

315 D-loop Ins C 36

315 D-loop Del C* 1

316 D-loop G→A* 1

317 D-loop C→T* 2

321 D-loop T→G* 5

324 D-loop C→G* 3

325 D-loop C→T* 1

333 D-loop T→C* 1

343 D-loop C→T*5

345 D-loop C→T* 4

349 D-loop C→A 3

366 D-loop G→A 6

369 D-loop C→A 1

371 D-loop C→T* 1

380 D-loop G→C 4

389 D-loop G→A 5

411 D-loop C→G 5

414 D-loop T→G* 5

424 D-loop T→A* 2

431 D-loop C→A 2

440 D-loop A→C 2

445 D-loop C→A 4

447 D-loop C→A**** 5

460 D-loop T→C* 4

477 D-loop T→C* 2

481 D-loop C→T* 3

483 D-loop C→T 5

484 D-loop A→C 1

489 D-loop T→C* 4

499 D-loop G→A* 1

505 D-loop C→T 4

509 D-loop C→A 5

512 D-loop A→C* 1

513 D-loop G→A* 5

516 D-loop C→A 6

518 D-loop C→A 9

518 D-loop Ins AC* 2

518 D-loop Ins AA 2

519 D-loop A→G ** 2

519 D-loop Del A 2

520 D-loop Del C* 4

522 D-loop Del C* 6

522 D-loop C→A 3

523 D-loop Del A* 28

524 D-loop Del C* 22

527 D-loop C→T*** 2

530 D-loop C→T* 3

538 D-loop A→G* 1

545 D-loop G→A 2

548 D-loop C→A 7

549 D-loop C→A 3

574 D-loop A→C* 3

573 D-loop Ins C 5

578 tRNA Phe T→G 7

582 tRNA Phe T→A 3

611 tRNA Phe Ins G *** 2

622 tRNA Phe G→A 3

627 tRNA Phe Del G 14

641 tRNA Phe A→T*** 1

652 12S rRNA G→A 3

652 12S rRNA G→T 1

653 12S rRNA Del G 8

659 12S rRNA T→G 4

660 12S rRNA C→G 7

664 12S rRNA G→C 1

686 12S rRNA Del A 25

686 12S rRNA A→G 3

687 12S rRNA G→A* 9

688 12S rRNA Del A 3

689 12S rRNA T→A 10

689 12S rRNA T→G* 1

690 12S rRNA T→A 4

690 12S rRNA Del T 5

693 12S rRNA A→C 5

694 12S rRNA C→A 4

695 12S rRNA A→G 5

696 12S rRNA T→C 3

697 12S rRNA Del G 3

698 12S rRNA Del C 3

699 12S rRNA Del A 3

700 12S rRNA Del A 19

702 12S rRNA C→A 2

708 12S rRNA Del C 14

708 12S rRNA C→G 6

709 12S rRNA G→A* 7

709 12S rRNA Del G 5

710 12S rRNA T→C* 1

712 12S rRNA C→T 17

716 12S rRNA T→G*** 5

720 12S rRNA Del T 6

721 12S rRNA Del T 12

721 12S rRNA T→C 6

722 12S rRNA C→T* 4

723 12S rRNA A→C 4

723 12S rRNA A→G* 1

726 12S rRNA Del C 8

727 12S rRNA T→C 6

728 12S rRNA C→T*** 7

732 12S rRNA A→T 6

733 12S rRNA T→A 3

734 12S rRNA C→A 2

735 12S rRNA A→C 12

737 12S rRNA Del C 19

738 12S rRNA A→C 7

738 12S rRNA Del A 6

739 12S rRNA Del C 6

747 12S rRNA A→G 6

747 12S rRNA Del A 44

748 12S rRNA Del G 24

752 12S rRNA Del C 9

753 12S rRNA Del A 10

754 12S rRNA A→G 12

755 12S rRNA Del G 3

757 12S rRNA A→T*** 5

761 12S rRNA Del A 18

761 12S rRNA A→G 10

762 12S rRNA Del G 4

763 12S rRNA Del C 2

766 12S rRNA Del G 8

768 12S rRNA A→G 4

769 12S rRNA G→C 9

772 12S rRNA A→T* 10

773 12S rRNA Del T 7

780 12S rRNA Del C 7

851 12S rRNA A→T 2

853 12S rRNA C→A 3

855 12S rRNA A→T 2

867 12S rRNA C→T 2

876 12S rRNA Del T 45

879 12S rRNA T→C* 4

908 12S rRNA Del C 2

910 12S rRNA A→T 2

912 12S rRNA Del T 15

913 12S rRNA Del A 16

914 12S rRNA A→C 2

917 12S rRNA Ins C 5

930 12S rRNA G→A* 4

932 12S rRNA Del C 3

933 12S rRNA Del G 3

949 12S rRNA Del T 13

951 12S rRNA G→A* 2

960 12S rRNA Ins C 10

960 12S rRNA Ins T 5

960 12S rRNA Ins CT 2

961 12S rRNA T→C* 3

961 12S rRNA Ins TC 2

965 12S rRNA Ins C 24

978 12S rRNA Del A 6

979 12S rRNA C→T* 2

984 12S rRNA C→G  **16**

984 12S rRNA Del C **58**

1018 12S rRNA G→A* 19

1048 12S rRNA C→T* 2

1189 12S rRNA T→C* 3

1243 12S rRNA T→C* 1

1303 12S rRNA G→A* 2

1387 12S rRNA A→T 8

1406 12S rRNA T→C* 1

1420 12S rRNA T→C* 3

1437 12S rRNA T→C 17

1438 12S rRNA A→G* **95**

1592 12S rRNA T→C 10

1609 tRNA val T→C 43

1614 tRNA val Del T 38

1631 tRNA val C→A 7

1632 tRNA val T→G 16

1633 tRNA val T→G 16

1633 tRNA val Del T 4

1638 tRNA val T→A 34

1639 tRNA val T→G 15

1646 tRNA val T→G 9

1647 tRNA val T→C 10

1648 tRNA val Del T **93**

1652 tRNA val Del C 12

1653 tRNA val T→A **76**

1653 tRNA val Del T **20**

1654 tRNA val T→A 5

1657 tRNA val Del C 11

1658 tRNA val T→C 22

1658 tRNA val T→G 2

1659 tRNA val Del T **89**

1662 tRNA val C→G 3

1664 tRNA val G→A* 1

1667 tRNA val Del C 5

1667 tRNA val C→A 17

1668 tRNA val T→A 8

1668 tRNA val Del T 3

1670 tRNA val A→C 17

7420 COX1 Del A 12

7421 COX1 Del A 46

7421 COX1 A→G 15

7422 COX1 Del G 45

7422 COX1 G→A 4

7423 COX1 Del A 9

7423 COX1 A→G 39

7424 COX1 A→T 6

7424 COX1 Del A 31

7450 tRNA Ser Ins C 6

7450 tRNA Ser Del A 7

7450 tRNA Ser Ins A 4

7450 tRNA Ser A→C 6

7518 tRNA Asp A→G 1

7521 tRNA Asp G→A* 15

8036 COX2 Del C 5

8091 COX2 Ins G 5

8099 COX2 Del A 3

8156 COX2 Del G 7

8156 COX2 Ins G 2

8187 COX2 Del G 4

8206 COX2 G→A* 2

8219 COX2 G→A 2

8221 COX2 Del A 7

8221 COX2 A→T 5

8223 COX2 Del T 6

8224 COX2 Del A 4

8225 COX2 Del A 12

8226 COX2 Del T 6

8231 COX2 Del C 6

8231 COX2 C→T 3

8236 COX2 Del A 13

8237 COX2 Del A **68**

8246 COX2 Del A 22

8251 COX2 G→A* 3

8254 COX2 Del C 15

8260 COX2 Del T 25

8260 COX2 T→A 4

8261 COX2 Del A 20

8262 COX2 C→A 20

8262 COX2 Del C 3

8264 COX2 Ins C 11

8264 COX2 C→T* 5

8265 COX2 Del T 5

8267 COX2 Del T 9

8269 COX2 G→C 4

8269 COX2 G→A* 5

8275 NC7 Del C 10

8275 NC7 Ins C 2

8276 NC7 Del C 42

8276 NC7 Del C 3

8285 NC7 Del C 41

8285 NC7 C→T 4

8285 NC7 C→A* 2

8295 tRNA Lys Del C 36

8303 tRNA Lys Del A 17

8308 tRNA Lys Del A 47

8308 tRNA Lys A→C 10

8319 tRNA Lys Del A 26

8319 tRNA Lys A→C 10

8320 tRNA Lys Del C 3

8321 tRNA Lys C→T 3

8321 tRNA Lys Del C 3

8322 tRNA Lys T→C 3

8325 tRNA Lys Del T 3

8326 tRNA Lys Del A 3

8326 tRNA Lys A→T 3

8468 ATPase8 C→T* 3

8472 ATPase8 C→G 1

8473 ATPase8 T→G 3

8473 ATPase8 T→C* 2

8474 ATPase8 C→T* 1

8476 ATPase8 C→T 2

8477 ATPase8 T→C 2

8485 ATPase8 Del G 3

8486 ATPase8 Del C 2

8502 ATPase8 Del A 2

8503 ATPase8 Del T 3

8507 ATPase8 A→C 1

8512 ATPase8 Del A 2

8513 ATPase8 Del C 2

8515 ATPase8 Del C 3

8519 ATPase8 G→A 2

8527 ATPase8 A→G* 1

8532 ATPase8 Del C 3

8539 ATPase8 Del C 4

8557 ATPase8 G→A* 1

8563 ATPase8 Del A 1

8566 ATPase8 A→G 2

8655 ATPase6 C→T* 4

8697 ATPase6 G→A* 3

8700 ATPase6 A→G 3

8701 ATPase6 A→G* 22

8836 ATPase6 A→G* 1

8860 ATPase6 A→G* **96**

8866 ATPase6 Ins A 9

8875 ATPase6 T→A 19

8875 ATPase6 Del T 16

8876 ATPase6 Del T 12

8877 ATPase6 Del T  **49**

8889 ATPase6 T→A **51**

8889 ATPase6 T→C **16**

8889 ATPase6 Del T **24**

8890 ATPase6 Del A 9

___________________________________________________________________

* Mitomap Reference.(includes mutations associated with different types of cancer)

** Himani Sharma, Archna Singh, Chandresh Sharma, Sunesh Kumar Jain and Neeta Singh. Mutations in the mitochondrial DNA D-loop region are frequent in cervical cancer.Cancer Cell Int. 2005 Dec 16;5:34.

***Cheng-Bo Han, Fan Li, Yu-Jie Zhao, Jia-Ming Ma, Dong-Ying Wu, Yu-Kui Zhang, Yan Xin .Variations of mitochondrial D-loop region plus downstream gene 12S rRNA-tRNA(phe) and gastric carcinomas. World J Gastroenterol. 2003 Sep;9(9):1925-9.

****Chen GF, Chan FL, Hong BF, Chan LW, Chan PS

Mitochondrial DNA mutations in chemical carcinogen-induced rat bladder and human bladder cancer. Oncol Rep. 2004 Aug;12(2):463-72.
